# Objective Hair Shaft Diameter Measurement From Trichoscopic Images Using Intensity Profile Analysis

**DOI:** 10.7759/cureus.114257

**Published:** 2026-08-10

**Authors:** Jesús Iván Martínez-Ortega, Frida Itzel Rosas-Lezama

**Affiliations:** 1 Histology, Autonomous University of Nuevo Leon, Monterrey, MEX; 2 Dermatology, Mexican Social Security Institute (IMSS), Piedras Negras, MEX; 3 Internal Medicine, Mexican Social Security Institute (IMSS), Merida, MEX

**Keywords:** anisotrichosis, biomedical image analysis, digital morphometry, female pattern hair loss, hair morphometry, hair shaft diameter, image analysis, imagej, quantitative trichoscopy, trichoscopy

## Abstract

Hair shaft diameter variability (anisotrichosis) is the most sensitive trichoscopic feature of androgenetic alopecia, yet its objective assessment remains dependent on manual measurements or dedicated image-analysis platforms. This technical report describes a simple and standardized workflow for objective hair shaft morphometry using grayscale intensity profile analysis from conventional trichoscopic images. A trichoscopic image obtained from a 50-year-old woman with female pattern hair loss was analyzed using Fiji (ImageJ). After grayscale conversion and threshold-based binarization, the image was systematically divided into four quadrants, and five free-standing proximal hair shafts were selected from each quadrant. Perpendicular grayscale intensity profiles were generated using the native Plot Profile function, and hair shaft width was estimated by counting consecutive low-intensity profile points below a predefined grayscale threshold of 200 arbitrary units, which was empirically selected for this proof-of-concept analysis. Twenty individual hair shafts were successfully analyzed. The proposed workflow consistently generated well-defined grayscale intensity profiles that enabled objective estimation of hair shaft width using only native Fiji functionality without proprietary software, additional plugins, or programming expertise. This single-image proof-of-concept study demonstrates how native image-analysis functions can be repurposed to transform qualitative dermatologic observations into objective morphometric descriptors while establishing a methodological foundation for future automated hair morphometry and quantitative characterization of hair shaft heterogeneity.

## Introduction

Hair shaft diameter is one of the principal quantitative parameters evaluated during trichoscopy and contributes to the assessment of multiple hair disorders, including androgenetic alopecia (AGA), telogen effluvium, alopecia areata, and selected scarring alopecias. Among these conditions, hair shaft thickness heterogeneity (anisotrichosis) represents the hallmark trichoscopic feature of AGA because it reflects progressive and asynchronous follicular miniaturization. Clinically, anisotrichosis is defined as variation in hair shaft diameter exceeding approximately 20% in men and 10% in women and has been reported in approximately 94% of patients with AGA, making it the most sensitive trichoscopic finding of the disease. In women, its frequency increases progressively with disease severity, further emphasizing the importance of objective assessment of hair shaft caliber [1-4].

Objective assessment of hair shaft caliber has evolved considerably over recent decades. Current approaches include manual border-to-border measurements performed on trichoscopic images, quantitative trichoscopic analysis using computer-assisted systems, phototrichograms, and dedicated commercial image-analysis platforms capable of estimating hair diameter and hair density. Although these techniques provide quantitative information, they frequently require specialized software, proprietary systems, or substantial operator interaction, limiting their accessibility in routine clinical practice and resource-limited settings. Consequently, there remains a need for simple, transparent, and standardized image-analysis workflows capable of generating quantitative hair shaft measurements using widely available software [5-8].

Among freely available biomedical image-analysis platforms, Fiji (ImageJ) has become one of the most extensively used environments for quantitative image processing because of its comprehensive collection of native analytical tools and broad applicability across biomedical disciplines. Building upon previous applications of Fiji for quantitative dermatologic image analysis, including objective assessment of color heterogeneity and lesion border morphology, the present work extends intensity-based image analysis to quantitative trichoscopic hair shaft evaluation [9-11]. One of Fiji's native analytical functions, Plot Profile, converts grayscale intensity values sampled along a user-defined line into intensity profiles that objectively characterize structural transitions within an image. Although intensity profile analysis has been widely employed in microscopy and biomedical image analysis for dimensional measurements, to our knowledge it has not yet been systematically adapted and documented for quantitative trichoscopic hair shaft morphometry [12].

Accordingly, this technical report presents a single-image proof-of-concept workflow illustrating how grayscale intensity profile analysis can be used to obtain image-derived estimates of hair shaft width from conventional trichoscopic images. Rather than providing clinical validation, the objective is to establish and document a simple, transparent, and standardized methodological framework that can be implemented using only native Fiji functions without proprietary software, additional plugins, or programming expertise. Although calibration and clinical validation remain subjects for future investigation, the proposed workflow provides an accessible methodological foundation for future quantitative trichoscopy, automated image analysis, and objective characterization of hair shaft heterogeneity.

## Technical report

Methods

A trichoscopic image was obtained from a 50-year-old woman with clinically and dermoscopically confirmed female pattern hair loss demonstrating characteristic anisotrichosis on the vertex scalp. The patient was not receiving treatment for hair loss at the time of image acquisition. The image showed natural dark hair without visible hair dye, significant gray hair, or inflammatory scalp changes. The image was originally acquired during routine clinical evaluation and was retrospectively selected to illustrate the proposed methodological workflow. Images were obtained using a DermLite DL4 dermatoscope and imported into Fiji (ImageJ) in their original JPEG format without modification of image dimensions.

**Figure 1:**
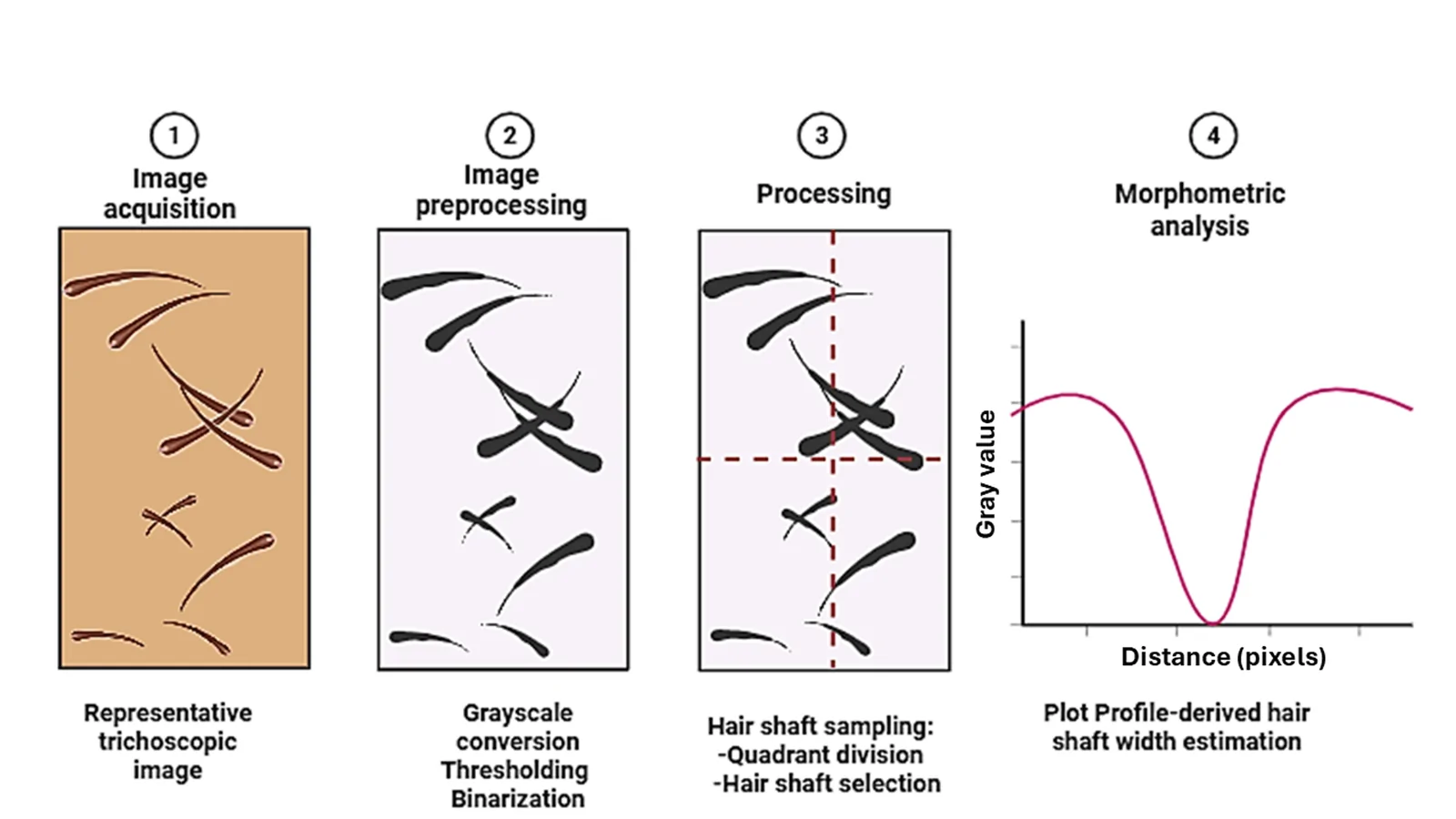
Methodological workflow for objective hair shaft morphometry using grayscale intensity profile analysis. The workflow illustrates trichoscopic image acquisition, image preprocessing, systematic hair shaft sampling, and Plot Profile-based estimation of hair shaft width. The schematic was designed and manually created by the authors using BioRender.com. No artificial intelligence-assisted image generation was used.

The overall image-analysis workflow is illustrated in Figure 1. The acquired image was imported into Fiji (ImageJ), converted to 8-bit grayscale using the native Image > Type > 8-bit function, and subsequently thresholded and binarized. The grayscale image was used for Plot Profile analysis, whereas the binary image facilitated visualization of hair shaft boundaries during image processing. The threshold of 200 arbitrary grayscale units was empirically selected from the grayscale intensity characteristics of the analyzed image to approximate the transition between background and hair shaft. This threshold is image- and acquisition-dependent and should not be interpreted as a universal value.

**Figure 2:**
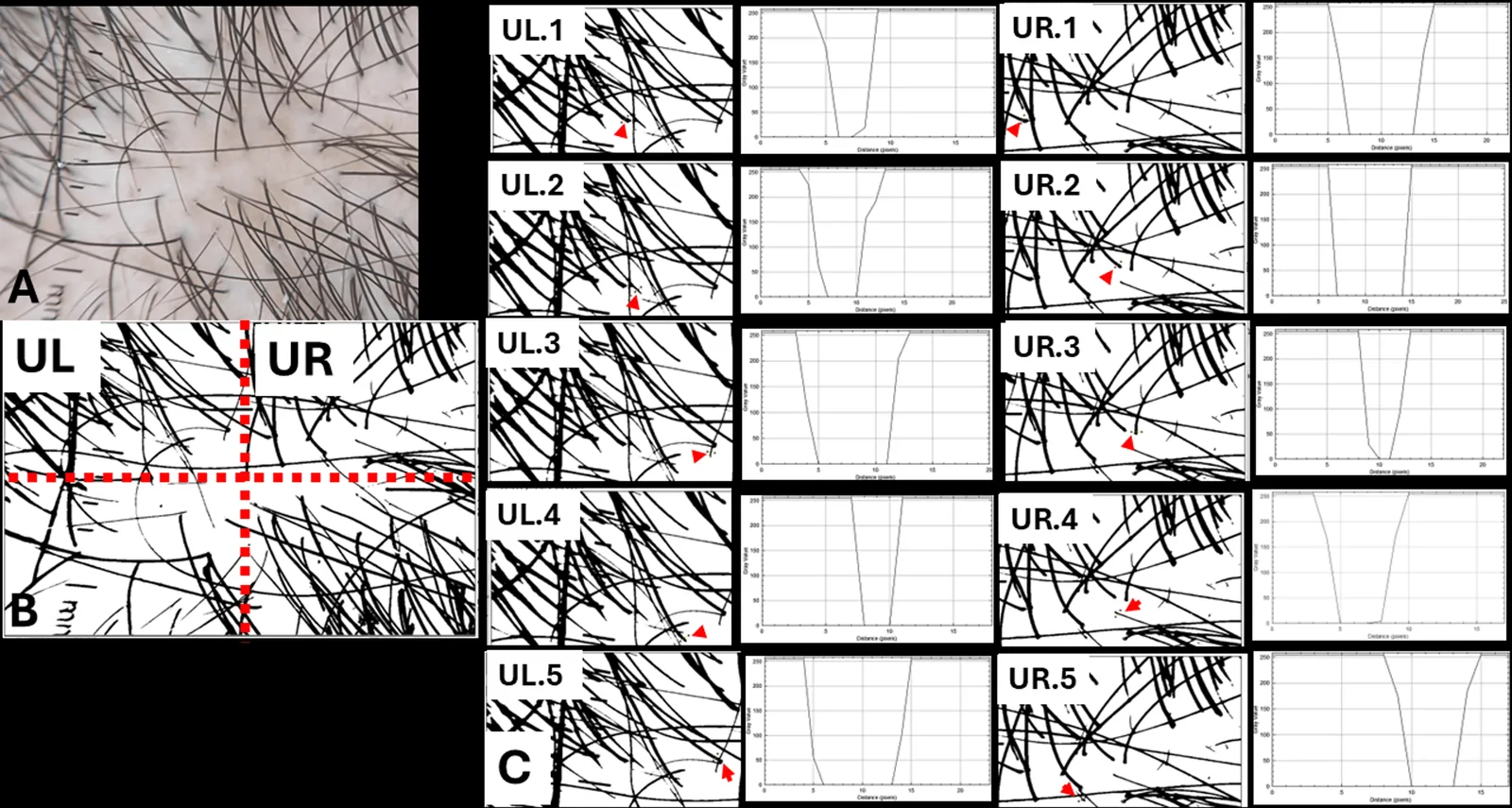
Representative Plot Profile-derived hair shaft width measurements from the upper image quadrants. (A) Original trichoscopic image obtained with a DermLite DL4 dermatoscope. (B) Image after grayscale conversion, thresholding, binarization, and systematic division into the upper left (UL) and upper right (UR) quadrants. (C) Representative proximal hair shaft selection and corresponding Plot Profile analyses from the upper scalp quadrants. Red arrowheads indicate the location where a perpendicular line was drawn across the proximal hair shaft to generate the corresponding Plot Profile. UL = upper left quadrant; UR = upper right quadrant. Numbers (e.g., UL.1 and UR.3) indicate the sequentially analyzed hair shaft within each quadrant. In the Plot Profile graphs, the x-axis represents Distance (pixels) and the y-axis represents Gray Value, as displayed by the native Fiji Plot Profile function. Panels A–C correspond to the practical implementation of the workflow illustrated in Figure 1, where Panel A represents Step 1 (image acquisition), Panel B represents Steps 2–3 (image preprocessing and processing), and Panel C represents Steps 3–4 (hair shaft selection and morphometric analysis using Plot Profile).

**Figure 3:**
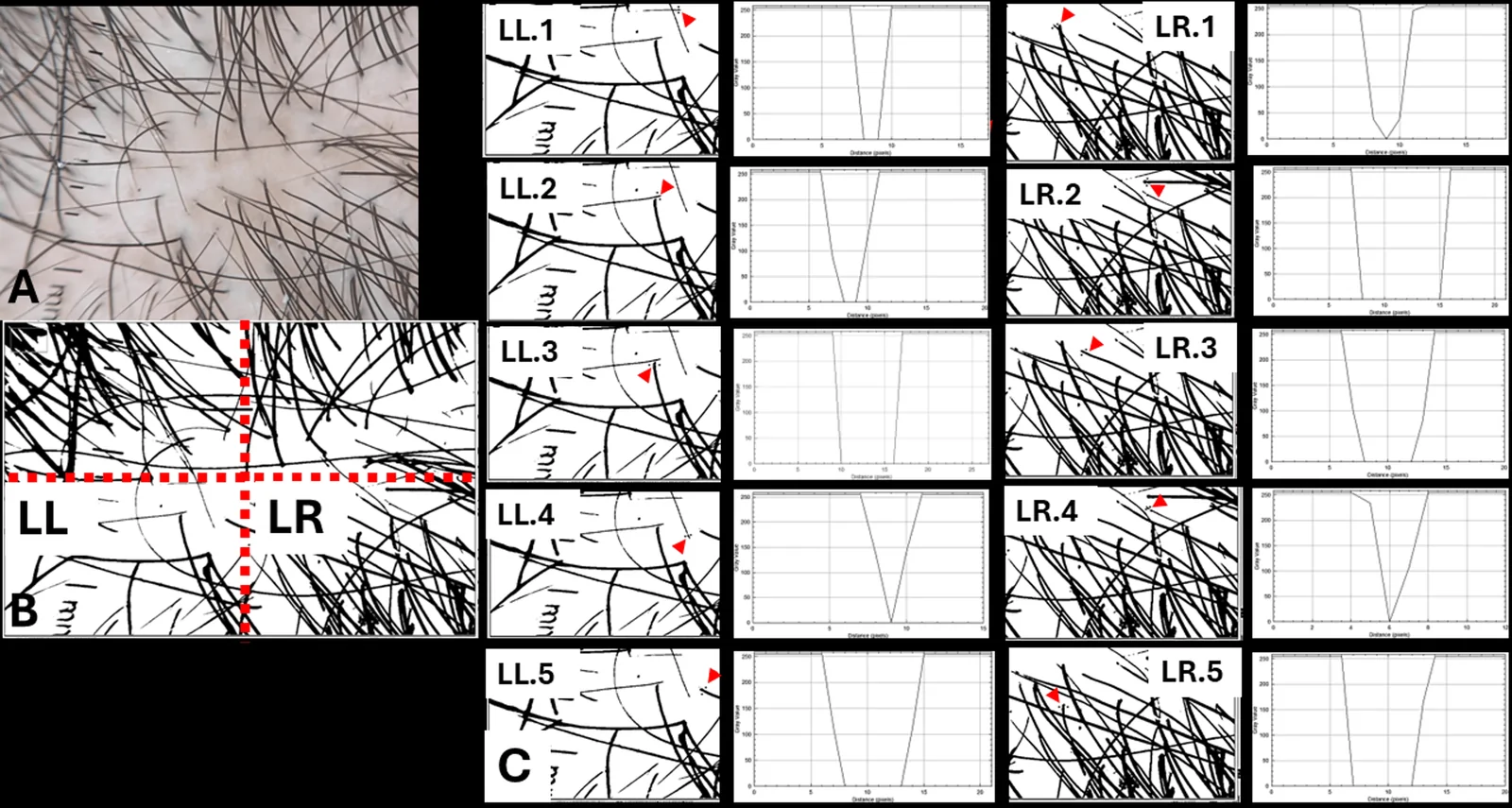
Representative intensity profile analysis from the lower image quadrants. (A) Original trichoscopic image obtained with a DermLite DL4 dermatoscope. (B) Image after grayscale conversion, thresholding, binarization, and systematic division into the lower left (LL) and lower right (LR) quadrants. (C) Representative proximal hair shaft selection and corresponding Plot Profile analyses from the lower scalp quadrants. Red arrowheads indicate the location where a perpendicular line was drawn across the proximal hair shaft to generate the corresponding Plot Profile. LL = lower left quadrant; LR = lower right quadrant. Numbers (e.g., LL.2 and LR.5) indicate the sequentially analyzed hair shaft within each quadrant. In the Plot Profile graphs, the x-axis represents Distance (pixels) and the y-axis represents Gray Value, as displayed by the native Fiji Plot Profile function. Panels A–C correspond to the practical implementation of the workflow illustrated in Figure 1, where Panel A represents Step 1 (image acquisition), Panel B represents Steps 2–3 (image preprocessing and processing), and Panel C represents Steps 3–4 (hair shaft selection and morphometric analysis using Plot Profile).

To promote standardized sampling and reduce operator-dependent selection bias, the image was divided into four equal quadrants (upper left, upper right, lower left, and lower right) by horizontal and vertical lines passing through the geometric center of the image. Hair shafts were selected according to predefined eligibility criteria. Five free-standing proximal hair shafts were selected from each quadrant, preferentially choosing centrally located shaft segments immediately distal to the follicular opening. Hair shafts intersected by adjacent shafts, partially obscured, or unsuitable for perpendicular profile acquisition were excluded. Selection proceeded sequentially from left to right within each quadrant (Figures 2, 3). All quadrants contained at least five eligible hair shafts; therefore, no replacement strategy was required.

For each selected hair shaft, a straight line was manually positioned perpendicular to the shaft axis. Grayscale intensity values were extracted using the native Plot Profile function in Fiji. Hair shaft width was estimated as the number of consecutive profile points presenting grayscale intensity values below the predefined threshold of 200 arbitrary units.

Twenty individual Plot Profiles were analyzed, and the corresponding hair shaft width estimates were recorded (Table 1). Because this study was designed as a single-image proof-of-concept technical report, no assessment of interobserver agreement, repeatability, or calibration against physical measurements was performed.

**Table 1:** Hair shaft width estimation obtained from Plot Profile analysis. Hair shaft width measurements obtained from grayscale intensity profile analysis. Hair shaft width was estimated by counting consecutive Plot Profile points with grayscale intensity values below a predefined threshold of 200 arbitrary units. Start and end points indicate the first and last consecutive profile points below the threshold, respectively.

Hair	Quadrant	First <200	Last <200	Width (profile points)
1	UL	5	8	4
2	UL	5	12	8
3	UL	8	10	3
4	UL	4	12	9
5	UL	4	13	10
6	UR	7	15	9
7	UR	7	14	8
8	UR	10	13	4
9	UR	5	10	6
10	UR	10	15	6
11	LL	8	9	2
12	LL	7	10	4
13	LL	10	16	7
14	LL	8	10	3
15	LL	7	14	8
16	LR	8	12	5
17	LR	8	15	8
18	LR	7	13	7
19	LR	6	8	3
20	LR	7	13	7

Results

The proposed workflow was completed for all 20 individual hair shafts selected according to the predefined eligibility criteria.

Plot Profile analysis consistently generated well-defined grayscale intensity valleys corresponding to individual hair shafts. Hair shaft width was estimated by counting consecutive profile points with grayscale intensities below the predefined threshold of 200 arbitrary units.

Hair shaft width estimates ranged from 2 to 10 profile points, with a median of 6.5 profile points and an interquartile range (Q1-Q3) of 4-8 profile points (IQR = 4) (Table 2), indicating moderate variability in image-derived hair shaft width estimates within the analyzed trichoscopic field. Individual measurements for all analyzed hair shafts are summarized in Table 1, whereas representative Plot Profiles obtained from the upper and lower image quadrants are presented in Figures 2, 3.

**Table 2:** Descriptive statistics of hair shaft width estimates. Descriptive statistics of hair shaft width estimates derived from grayscale intensity profile analysis. Width values are expressed as profile points, representing relative image-derived measurements rather than calibrated physical dimensions.

Statistic	Value
Number of analyzed hair shafts	20
Minimum	2 profile points
Maximum	10 profile points
Median	6.5 profile points
Interquartile range (IQR)	4 profile points (Q1 = 4; Q3 = 8)

Hair shaft width, expressed as profile points, is an image-specific, uncalibrated relative measurement rather than a physical unit such as micrometers. Therefore, these values should not be interpreted as absolute hair shaft diameters or directly compared across images acquired under different imaging conditions.

The complete workflow, from trichoscopic image acquisition to grayscale intensity profile analysis and image-derived hair shaft width estimation, is summarized in Figure 1.

The proposed methodology relied exclusively on native Fiji functionality and required neither dedicated trichoscopic software, proprietary image-analysis platforms, additional plugins, automated segmentation algorithms, nor programming expertise, thereby maintaining a simple and accessible workflow for proof-of-concept quantitative trichoscopic image analysis.

## Discussion

Hair shaft diameter variability (anisotrichosis) is considered the most sensitive trichoscopic feature of AGA and reflects the underlying process of progressive follicular miniaturization. Although objective assessment of hair shaft caliber has become increasingly important for diagnosis and disease monitoring, quantitative evaluation in routine clinical practice remains limited by manual measurements and dependence on proprietary image-analysis platforms or dedicated trichoscopic software [1,2,6-8]. The present single-image proof-of-concept study demonstrates that native image-analysis functions can, in principle, be adapted to generate image-derived hair shaft morphometry from conventional trichoscopic images. However, the present work establishes methodological feasibility rather than clinical performance or generalizability.

The proposed workflow estimates hair shaft width from grayscale intensity transitions rather than from direct visual identification of shaft borders. This intensity-based approach provides a predefined measurement framework while preserving methodological transparency and requiring only freely available software. Because the workflow relies exclusively on native Fiji functions, it can be readily implemented without proprietary image-analysis platforms, additional plugins, or programming expertise. A predefined sampling strategy - including quadrant division, eligibility-based hair shaft selection, and perpendicular intensity profile acquisition - was incorporated to promote methodological consistency and reduce operator-dependent selection bias. Although manual shaft selection remains necessary, the measurements are derived from predefined grayscale intensity information rather than subjective border identification. Because the present study did not compare this workflow with conventional border-to-border measurements or commercial quantitative trichoscopic systems, no conclusions regarding superiority or reduced measurement subjectivity should be inferred. Likewise, software accessibility should not be interpreted as evidence of measurement reproducibility, which requires formal validation [12].

The descriptive statistics summarized in Table 2 provide a concise quantitative overview of the image-derived hair shaft width estimates obtained using the proposed workflow. The observed median and interquartile range describe the distribution of measurements within the analyzed trichoscopic field and are consistent with the expected heterogeneity of hair shaft caliber in female pattern hair loss. Nevertheless, because the measurements are expressed as image-specific profile points rather than calibrated physical dimensions, these values should be interpreted as relative morphometric descriptors within the analyzed image rather than absolute estimates of hair shaft diameter.

The present work was intentionally designed as a methodological proof-of-concept rather than a clinical validation study. Accordingly, the objective was not to establish diagnostic thresholds, compare diagnostic performance, or evaluate discrimination between healthy and diseased hair. Instead, this study demonstrates the feasibility of obtaining image-derived hair shaft width estimates using a simple, transparent methodology based exclusively on native Fiji functionality [12]. Consequently, no conclusions regarding diagnostic thresholds, diagnostic accuracy, disease discrimination, or routine clinical applicability can be drawn from the present results. These questions require future studies involving calibrated measurements, multiple observers, independent patient cohorts, and comparisons with established quantitative trichoscopic techniques.

Rather than representing a software-specific solution, the proposed workflow illustrates a broader methodological principle in dermatologic image analysis: clinically relevant morphologic structures can be transformed into standardized quantitative signals from which image-derived morphometric descriptors can be extracted. Although demonstrated here using hair shafts, this conceptual framework is not restricted to trichoscopy and may facilitate development of additional quantitative image-analysis methodologies for other dermatologic conditions. Nevertheless, its broader applicability remains hypothetical and will require condition-specific validation before clinical implementation [9-11].

Limitations

Several limitations should be acknowledged. First, this technical report was intentionally designed as a single-image proof-of-concept study and therefore included only one representative trichoscopic image from a patient with female pattern hair loss. Consequently, no conclusions regarding diagnostic thresholds, diagnostic performance, clinical applicability, or disease discrimination can be drawn from this report.

Second, hair shaft selection remained operator-dependent because free-standing proximal hairs were manually identified before intensity profile analysis according to predefined eligibility criteria.

Third, hair shaft width was expressed as profile points rather than calibrated physical units. Consequently, the measurements represent image-specific relative morphometric descriptors and cannot be directly compared across different patients, imaging sessions, or imaging devices without appropriate calibration.

Finally, the proposed workflow remains semi-manual and has not yet undergone formal validation through calibration, intraobserver and interobserver reproducibility studies, comparison with manual border-to-border measurements, commercial quantitative trichoscopic systems, or automated image-analysis approaches.

Future directions

Future work should first focus on image calibration, followed by formal validation through intra- and interobserver reproducibility studies, comparison with established quantitative trichoscopic measurement techniques, and evaluation across multiple images, acquisition conditions, and patient populations. Automation of hair shaft detection and Plot Profile extraction would substantially improve efficiency while reducing operator intervention.

Despite these limitations, the present study establishes the methodological feasibility of transforming grayscale intensity profiles into quantitative, image-derived morphometric descriptors using a simple, transparent workflow. This proof-of-concept provides a practical foundation upon which future studies can incorporate calibration, reproducibility assessment, and clinical validation.

Beyond individual hair shaft measurements, future developments should move toward quantitative characterization of entire hair shaft width distributions. Distribution-based morphometric descriptors - including measures of central tendency, dispersion, and information entropy - may provide a more comprehensive mathematical representation of anisotrichosis than isolated diameter measurements alone. Validation of these descriptors across healthy individuals and different hair disorders may establish new quantitative biomarkers for objective trichoscopic assessment while further expanding the role of accessible quantitative image-analysis methodologies in dermatologic research.

## Conclusions

The present proof-of-concept study introduces a simple and transparent workflow for image-derived hair shaft morphometry using native grayscale intensity profile analysis from a single conventional trichoscopic image. Although demonstrated using Fiji, the proposed methodology is based on a broader image-analysis principle in which grayscale intensity transitions are transformed into quantitative morphometric descriptors that characterize clinically relevant structures.

Although further validation, calibration, and automation are required before routine clinical implementation, the proposed workflow establishes an initial methodological framework for accessible quantitative trichoscopy. More importantly, the principal contribution of the present work is not the implementation of a particular software tool but the demonstration that qualitative dermatologic image features can be systematically transformed into quantitative image-derived morphometric descriptors through standardized intensity profile analysis. Rather than representing a Fiji-specific solution, the present workflow illustrates a methodological principle that may prove useful for future quantitative dermatologic image analysis. Whether this framework can be generalized beyond the present proof-of-concept application will require validation in additional dermatologic imaging settings.
